# Enhancing the Performance of Ba_x_MnO_3_ (x = 1, 0.9, 0.8 and 0.7) Perovskites as Catalysts for CO Oxidation by Decreasing the Ba Content

**DOI:** 10.3390/nano14161334

**Published:** 2024-08-10

**Authors:** Á. Díaz-Verde, M. J. Illán-Gómez

**Affiliations:** Carbon Materials and Environment Research Group, Inorganic Chemistry Department, University of Alicante, Ctra San Vicente del Raspeig s/n, San Vicente del Raspeig, 03690 Alicante, Spain; alvaro.diaz@ua.es

**Keywords:** perovskite, barium, manganese, CO oxidation, oxygen vacancies

## Abstract

Mixed oxides featuring perovskite-type structures (ABO_3_) offer promising catalytic properties for applications focused on the control of atmospheric pollution. In this work, a series of Ba_x_MnO_3_ (x = 1, 0.9, 0.8 and 0.7) samples have been synthesized, characterized and tested as catalysts for CO oxidation reaction in conditions close to that found in the exhausts of last-generation automotive internal combustion engines. All samples were observed to be active as catalysts for CO oxidation during CO-TPRe tests, with Ba_0.7_MnO_3_ (B0.7M) being the most active one, as it presents the highest amount of oxygen vacancies (which act as active sites for CO oxidation) and Mn (IV), which features the highest levels of reducibility and the best redox properties. B0.7M has also showcased a high stability during reactions at 300 °C, even though a slightly lower CO conversion is achieved during the second consecutive reaction cycle. This performance appears to be related to the decrease in the Mn (IV)/Mn (III) ratio.

## 1. Introduction

Currently, the scientific community is focused on looking for solutions to minimize the emissions of polluting gasses, which come from, among other sources, the industrial and automotive sectors. In addition to CO_2_, which is one of the most problematic species in the automobile sector [1,2], CO is considered to be an anthropogenic toxic pollutant if its concentration exceeds the amount established by environmental regulations [3]. The catalyzed CO oxidation is the main strategy for the removal of CO from the automobile exhaust, which is one of the main current sources. Pt-based formulations are among the most effective catalysts [4,5,6] for CO oxidation. However, the use of noble metal-based catalysts presents two main drawbacks: (i) the high cost, due to their limited availability [7]; and (ii) the sintering at high temperatures [8,9], which causes a remarkable decline in the catalytic performance. Consequently, the development of affordable catalysts that could match the catalytic performance of noble metals but avoid the sintering problem is an urgent target. In recent years, several transition metal oxides have been identified as potential catalysts for CO oxidation, such as Co_3_O_4_, CuO, MnO_2_ and CeO_2_ [10,11,12,13].

In this sense, transition metal-based mixed oxides with perovskite structures (ABO_3_) are considered to be an interesting family of solids for, among other applications [14,15], oxidation reactions and, thus, also for catalytic CO oxidation [16]. This is because their structure allows for the stability of a huge amount of non-noble metal mixed oxides with the appropriate cationic radii to fit well in the A (12 coordination) and B (6 coordination) sites. Specifically, A-sites are usually occupied by rare earth, alkaline and alkaline–earth metals; meanwhile, transition metals commonly occupy the B-sites [17]. This structure also enables the partial substitution of A and/or B cations, leading to the creation of structural defects and a variable oxygen non-stoichiometry. These characteristics, as well as the high stability and redox properties, allow perovskite oxides to present a wide applicability in catalytic reactions. The redox processes of these solids, both on the surface and in the bulk, account for the reversible loss and uptake of oxygen and the creation and filling up of oxygen vacancies [16,18,19]. In fact, manganese-based perovskites have been proposed as convenient catalysts for CO oxidation [20], as this metal presents not only atomic orbitals with the appropriate symmetry and energy levels for the activation of CO and O_2_ molecules [21] but also a great versatility for modifying its oxidation state, thus favoring the redox processes [22,23]. In perovskites, the electronic configuration of B cation plays a crucial role in the catalytic performance for oxidation reactions, as it determines the adsorption and the activation of the reactants (CO and O_2_ in the context of CO oxidation). In fact, the occupancy of the e_g_ orbital, which presents the optimal geometry for interacting with the molecular orbitals of reactants, dictates the energies involved in the adsorption and the desorption of reactants [16], as the energy of adsorption increases as the number of electrons in the e_g_ orbital decreases. For Mn (III), an effective interaction with CO molecules exits, since three electrons are located in the t_2g_ orbitals and one is located in the e_g_ orbitals, so the partially empty e_g_ orbital accepts the pair of lone electrons from the carbon atom of CO while backdonation of a t_2g_ electron to the antibonding π^*^ orbital of the CO molecule occurs [21]. Conversely, Mn (IV) presents only three electrons in the t_2g_ orbitals (and lacks electrons in e_g_ orbitals), affecting its interaction with CO and O_2_ through the strength of the Mn-O bond. Thus, by adjusting the physicochemical properties of Mn-based perovskites, and hence for the Mn (IV)/Mn (III) ratio, it is possible to weaken the Mn-O bond, enhancing the catalytic activity for CO oxidation [24].

In a previous work developed by the authors [25], the advantages of employing a Ba-deficient perovskite formulation (Ba_0.7_MnO_3_) for the CO oxidation in simulated gasoline car exhaust conditions were established. In this paper, the purpose is to try to enhance the performance of Ba_0.7_MnO_3_ perovskite by determining the effect of the gradual decrease in Ba content in the catalytic activity of the BaMnO_3_ mixed oxide through the synthesis and characterization of a Ba_x_MnO_3_ (x = 1, 0.9, 0.8 and 0.7) series of perovskites to be used for CO oxidation in different conditions, simulating the composition of last-generation diesel and gasoline car exhausts. The detailed study of the gradual decrease in the Ba is of great importance, due to the possibility of finding optimal formulations with intermediate A cation contents, as reported in the literature [26].

## 2. Materials and Methods

The Ba_x_MnO_3_ (BxM) perovskites were synthesized using the sol–gel method adapted to an aqueous medium [27,28]. Briefly, first, a 40 mL solution (containing EDTA (C_10_H_16_N_2_O_8_, Sigma–Aldrich, St. Louis, MI, USA, 98.5% wt) used as a chelating agent in a 1:2 molar Mn:EDTA ratio) at 60 °C was prepared. Barium nitrate (Ba(NO_3_)_2_, Sigma–Aldrich, 99.0% wt) and manganese(II) nitrate tetrahydrate (Mn(NO_3_)_2_·4 H_2_O, Sigma–Aldrich, 99.0% wt) were added as metal precursors and, afterward, citric acid (C_6_H_8_O_7_, Sigma–Aldrich, 99.0% wt) was incorporated using the same molar ratio than for EDTA (1:2, Mn:citric acid). Subsequently, the temperature was increased to 80 °C for assuring the gel formation. During all the procedure, the pH was set at 9 by employing an ammonia solution (NH_3_, Panreac, Castellar del Valles, Spain, 30% wt). Finally, the obtained gel was dried at 150 °C during 12 h and, as the last step, the solid was calcinated at 850 °C for 6 h.

For a sample characterization, the following techniques have been used.

The surface composition of samples was determined by μ-XRF, employing an Orbis Micro-XRF Analyzer from EDAX.

The textural properties were determined by N_2_ adsorption at −196 °C using an Autosorb-6B instrument from Quantachrome (Anton Paar Austria GmbH, Graz, Austria). The samples were degassed at 250 °C for 4 h before the N_2_ adsorption experiments.

The crystalline structure was obtained using X-ray Diffraction (XRD). The X-ray patterns were recorded between 20° and 80° 2θ angles with a step rate of 0.4 °min^−1^ and using Cu K_α_ (0.15418 nm) radiation in a Bruker D8-Advance device. Average crystallite sizes [29] and lattice strain values [30] were determined employing the Williamson–Hall method. Finally, XRD refinement was performed to determine the percentage of the different crystalline phases in the sample using the HighScore Plus software (Malvern Panalytical B.V. Almelo, The Netherlands, 4.9 (4.9.0.27512) version).

The surface chemistry was evaluated by X-ray Photoelectron Spectroscopy (XPS) using a K-Alpha Photoelectron Spectrometer by Thermo-Scientific with an Al K_α_ (1486.7 eV) radiation source. To obtain the XPS spectra, the pressure of the analysis chamber was maintained at 5 × 10^−10^ mbar. The binding energy (BE) and kinetic energy (KE) scales were adjusted by setting the C 1s transition at 284.6 eV, and the BE and KE values were then determined with the peak-fit software of the spectrometer (Thermo Avantage v5.9929). To obtain the collected data, the following transitions were analyzed: O 1s, Mn 2p^3/2^, Mn 3p and Ba 3d^5/2^.

The reducibility of samples was estimated by Temperature-Programmed Reduction with H_2_ (H_2_-TPR) in a Pulse Chemisorb 2705 (from Micromeritics) provided with a Thermal Conductivity Detector (TCD) and using 30 mg of sample, which was heated at 10 °C min^−1^ from 25 °C to 1000 °C in 5% H_2_/Ar atmosphere (40 mL min^−1^). The quantification of the H_2_ consumption was carried out employing a copper (II) oxide (CuO, Sigma–Aldrich, 99.9% wt) reference sample, which is known to be reduced according to reaction (1) [31]. For the assignment of the H_2_ consumption peaks detected in the H_2_-TPR profiles, manganese (III) oxide (Mn_2_O_3_, Sigma–Aldrich, 99% wt) and manganese (IV) oxide (MnO_2_, prepared using the same manganese precursor and calcination conditions as those used for the perovskites synthesis, were employed as references, being then reduced according to reactions (2) and (3), respectively [32].

CuO + H_2_ → Cu + H_2_O (1)

Mn_2_O_3_ + H_2_ → 2MnO + H_2_O (2)

MnO_2_ + H_2_ → MnO + H_2_O (3)

O_2_-TPD experiments were performed in a Thermo Gravimetric Mass Spectrometrer (TG-MS) system (Q-600-TA and Thermostar from Balzers Instruments (Pfeiffer Vacuum GmbH, Aßlar, Germany) respectively), with 16 mg of sample heated at 10 °C min^−1^ from room temperature to 950 °C under a 100 mL/min of helium atmosphere. Before the experiments, each sample underwent a 1 h preheating treatment process at 150 °C to remove the moisture. During the experiments, the 32 *m*/*z* signal was followed for the O_2_ evolved during the experiments. The amount of evolved oxygen was also estimated using a CuO reference sample, which is decomposed to Cu_2_O under the tested conditions [33], according to reaction (4).

4CuO → 2Cu_2_O + O_2_ (4)

Temperature-Programmed Reduction with CO (CO-TPR) tests were carried out in order to obtain information about the conversion of CO into CO_2_ using exclusively the oxygen evolving from the perovskite samples. To obtain the CO-TPR profiles, the samples (50 mg of catalyst and 100 mg of SiC loaded into a U-shaped quartz reactor) were subjected to a heating at 10 °C min^−1^ until 600 °C in a 100 mL min^−1^ flow (Gas Hourly Space Velocity (GHSV) of 4967 h^−1^) of a 1% CO/He gas mixture [34]. Before starting the CO-TPR test, the mixture catalyst-SiC was preheated for 1 h at 700 °C in a 5% O_2_/He gas mixture for cleaning the surface of samples. Subsequently, an additional preheating (following the same conditions) was performed prior to the second CO-TPR cycle. For reaction product quantification, an Agilent 8860 gas chromatograph was used, provided with a TCD and two packed columns (Porapack-Q and MolSieve-13X (Agilent Technologies Spain, Madrid, Spain)). The CO conversion was calculated by employing Equation (1):

CO conversion (%) = ((CO_in_ − CO_out_)/CO_in_) · 100 (1)

where CO_out_ is the outlet molar flow rates of CO and CO_in_ is the inlet molar flow rate.

To determine the catalytic activity for CO oxidation, Temperature-Programmed Reaction (CO-TPRe) tests have been developed using three gas mixtures composed of the following: (i) 1% CO and 1% O_2_ in He, as an approximation to the CO partial pressure in the actual TWC working conditions [34]; (ii) 1% CO and 10% O_2_ in He, for analyzing the effect of using a higher oxygen partial pressure in relation to (i); and (iii) 0.1% CO and 1% O_2_ in He, for determining the effect on the catalytic performance in a very low CO partial pressure in relation to (i). For these experiments, the same reactor configuration and preheating treatment described for the CO-TPR tests were used with the addition of applying a 10 °C min^−1^ heating rate until 500 °C. Moreover, a commercial 1% Pt/Al_2_O_3_ sample was used as reference, which was not subjected to the preheating treatment in order to avoid Pt sintering [35]. Additionally, for the most active catalyst, a stability test consisting of two reaction cycles at 300 °C (5 h), using the gas mixture (i), was developed. Before each cycle, the catalyst was subjected to the corresponding pre-oxidation treatment. For reaction product quantification, the experimental setup described for CO-TPR tests was employed. The CO conversion was calculated using Equation (1).

## 3. Results and Discussion

### 3.1. Catalysts Characterization

#### 3.1.1. Chemical, Morphological and Structural Properties

Table 1 features the nomenclature, the BET surface area (calculated from N_2_ adsorption data), the chemical composition (determined by μ-XRF), and some relevant XRD data of BxM perovskites. The weight percentages follow the expected trend according to the decrease in Ba in the molecular formula and, as is usual for solids with an almost negligible porosity, these mixed oxides present very low surface areas [36,37].

**Figure 1:**
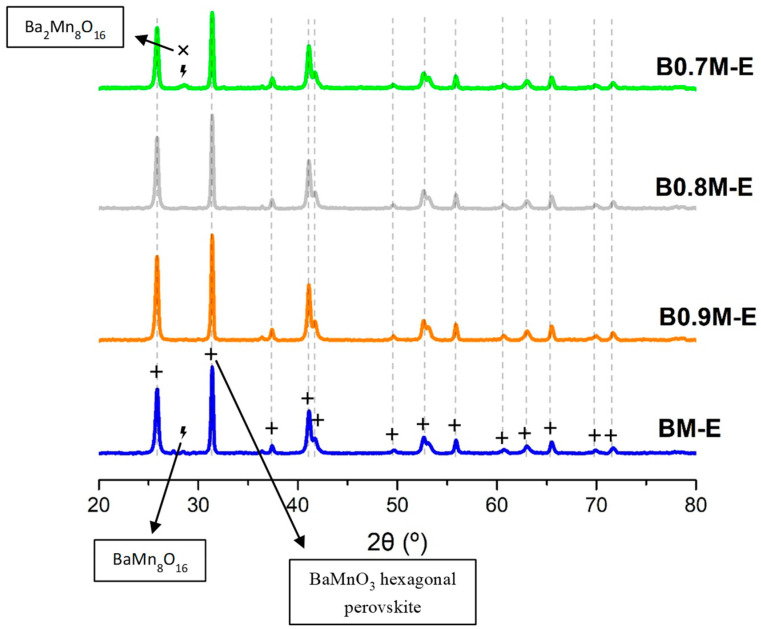
XRD patterns of BxM samples.

The X-ray patterns of the BxM samples shown in Figure 1 reveal the presence of the hexagonal BaMnO_3_ perovskite structure with a *P63/mmc* space group (JCPDS-ICDD 71-1594) as the main crystalline phase, and, as minor crystalline phases, two very low intensity peaks corresponding to BaMn_8_O_16_ (JCPDS-ICDD 29-188) and Ba_2_Mn_8_O_16_ (JCPDS-ICDD 78-962) have been detected. According to the following weight percentages obtained by XRD refinement: (i) for BM, BaMn_8_O_16_ contributes 1.6%; for B0.7M, BaMn_8_O_16_ and Ba_2_Mn_8_O_16_ contribute 3.6% and 3.9%, respectively. The contribution of these minor phases is very low. It should be noted that, as all the BaMnO_3_ diffraction peaks appear at the same 2θ value, a structural distortion due to the decrease in the barium content appears not to be taking place. Consequently, the cell parameters have not been modified (Table 1), and the lattice strain seems being alleviated with the decrease in the Ba content, especially for the intermediate compositions. However, the average crystallite size of the hexagonal perovskite phase is larger for BxM than for BM. This increase, which was also observed for La_0.4_Sr_0.4_Mn_x_Ti_1−x_O_3_ perovskites, appears to be attributed to the increase in the Mn percentage in the formulations [38].

#### 3.1.2. Surface Composition

**Figure 2:**
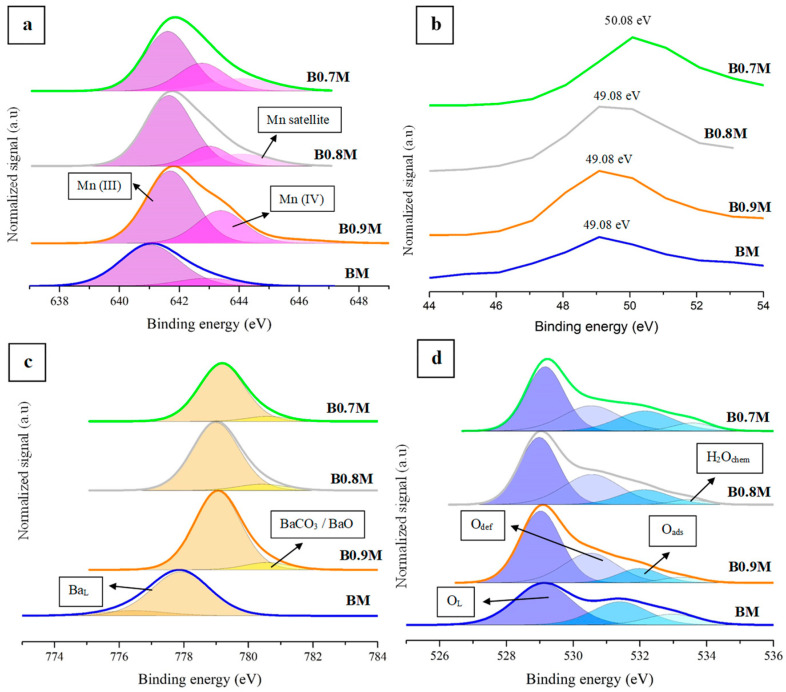
XPS spectra of the Mn 2p^3/2^ (**a**), Mn 3p (**b**), Ba 3d^5/2^ (**c**) and O 1s (**d**) transitions.

The XPS spectra of Ba, Mn, and O are shown in Figure 2a–c, being the corresponding XPS data summarized in Table 2.

In the Mn 2p^3/2^ spectra (Figure 2a), the three signals usually detected for Mn (III) and Mn (IV) species are featured, indicating the presence of these two oxidation states on the surface [39,40,41]. In these spectra, the binding energy of the deconvoluted band associated with Mn (III) is lower than that corresponding to Mn (IV), since the electronic charge density of the cation decreases as the oxidation state increases [39,40,41,42]. The values of the Mn (IV)/Mn (III) ratio (calculated based on the area of the deconvoluted bands) compiled in Table 2 indicate that, on the surface of all samples, a higher amount of Mn (III) than of Mn (IV) is present and the proportion of Mn (IV) increases as the Ba content decreases. In fact, the percentages of surface Mn (III) and Mn (IV) (Mn (III)_s_ and Mn (IV)_s_ shown in Table 2 and calculated based on the Mn (IV)/Mn (III) ratio and on the percentages of total Mn in the sample determined by μ-XRF, and they confirm that the amount of Mn (IV) increases as the Ba content decreases. This result clearly shows that the oxidation of Mn (III) to Mn (IV) takes place on the surface as an electronic charge compensation mechanism to counteract the decrease in the positive charge in the Ba-deficient perovskites. This effect was also observed in a series of La_1−x_Sr_x_MnO_3_ perovskites, where the substitution of La (III) by Sr (II) caused a positive charge deficiency [43], and in a BaMn_1−x_Cu_x_O_3_ perovskites series in a previous publication by the authors [44].

However, it is important to underline that, as in the Mn 2p^3/2^ spectra analyzed above, the binding energies of the Mn (III) and Mn (IV) deconvolutions are not significantly different, and so the accurate determination of the oxidation states of Mn is a difficult task. Thus, to try to ensure a correct assignment, the usefulness of the analysis of the splitting found in the Mn 3s transition has been proposed [42,45,46]. However, for the BxM samples, this analysis is hindered by the overlap of Mn 3s and Ba 4d transitions [45]. As an alternative, an Mn 3p transition also appears in the literature as an additional tool for determining the oxidation state of Mn [47], and the corresponding spectra for BxM samples are presented in Figure 2b. According to J.M. Cerrato et al. [47], a Mn 3p signal close to 49 eV is detected if Mn (III) is present as the main oxidation state on the catalyst surface. So, as observed in Figure 2b, Mn (III) appears to be confirmed as the main one for BxM samples. Moreover, the B0.7M spectrum shows a shift in the Mn 3p signal towards higher binding energies, indicating a higher amount of Mn (IV) [47] compared to other samples, and this confirms the conclusion extracted based on the values of the Mn (IV)/Mn (III) ratio calculated from the data of Mn 2p^3/2^ transition.

The Ba 3d^5/2^, presented in Figure 2c, shows two signals: (i) lattice Ba at lower binding energies and (ii) barium carbonate (formed due to the air exposition [48,49] of samples) and barium oxide at higher binding energies [50]. As could be expected, lattice barium is the main species in all BxM samples.

Finally, the deconvolution of the O 1s signal, featured in Figure 2d, reveals the presence of the different contributions usually found in perovskites, which are as follows: (i) a band located at binding energies around 529 eV, associated with the presence of lattice oxygen (O_L_) [51]; (ii) a band located around 531 eV, related to the existence of oxygen with low oxygen coordination that corresponds to the oxygen vacancies formed on the surface (O_def_) [52]; (iii) a signal at 532 eV, which indicates the presence of adsorbed oxygen, hydroxyl and carbonate groups on the catalyst surface (O_ads_) [53,54,55]; and (iv) a band located at approximately 533 eV, which corresponds to chemisorbed water (H_2_O_chem_) [56,57]. In Table 2, the XPS and nominal values of the O_L_/(Ba + Mn) ratio for all samples are shown. The experimental values have been obtained from the area under the deconvoluted signals and the nominal ones (between parentheses), calculated from the molecular formula. It is important to underline that an experimental ratio lower than the nominal one indicates the presence of oxygen vacancies on the surface. Thus, all samples present oxygen vacancies on the surface that increase for the Ba-deficient perovskites. This finding suggests that these oxygen vacancies are being generated as an additional charge compensation mechanism to counteract the positive charge defect caused by the decrease in the Ba content.

Thus, the analysis of the surface chemistry of the BxM perovskites reveals that two charge compensation mechanisms appear to be taking place to balance the positive charge deficiency caused by the low amounts of Ba: the oxidation of Mn (III) to Mn (IV) and the generation of oxygen vacancies.

#### 3.1.3. Redox Properties

The reducibility and the redox properties of samples were explored by developing Temperature-Programmed Reduction with H_2_ (H_2_-TPR), Temperature-Programmed Reduction with CO (CO-TPR) and Temperature-Programmed Desorption of O_2_ (O_2_-TPD) tests, as described in the experimental section.

**Figure 3:**
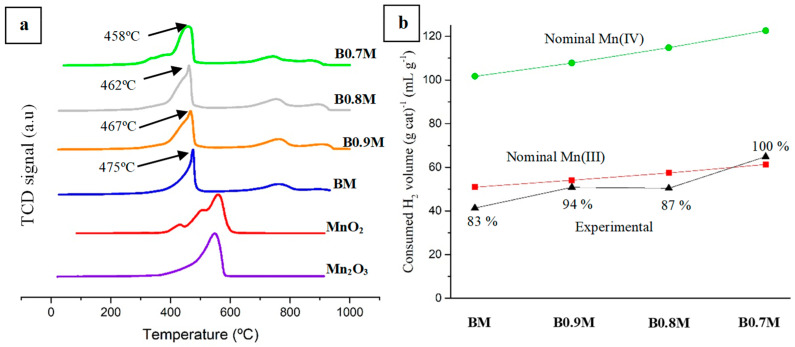
H_2_-TPR consumption profiles for BxM samples, for Mn_2_O_3_ and MnO_2_ references (**a**), and for H_2_ consumption (mL H_2_ (g of cat)^−1^) (**b**).

Regarding H_2_-TPR tests, the H_2_ consumption profiles for BxM samples, along with those corresponding to Mn_2_O_3_ and MnO_2_ used as references, are shown in Figure 3a. For perovskite samples, three peaks are usually identified [58,59,60,61]: (i) a low temperature peak, which appears between 400 °C and 500 °C, assigned to Mn (IV)/Mn (III) being reduced to Mn (II); (ii) a low intensity signal, at temperatures between 700 °C and 800 °C, corresponding to the reduction of oxygen species; and (iii) another very low intensity band at around 900 °C, due to the reduction of bulk Mn (III) to Mn (II). It is noteworthy that the Mn (IV) reduction to Mn (III) is also shown as a low intensity shoulder at lower temperatures than those corresponding to the main peak [62,63]. Since the intensity of this shoulder increases as the barium content decreases, it is suggested that the amount of Mn (IV) in the bulk increases with the decrease Ba percentage, as was previously concluded for the surface based on the XPS data (see Table 2). Focusing the attention on the maximum of the main reduction peak (corresponding to the reduction of Mn (IV)/Mn (III) to Mn (II)), whose temperature values are included in Figure 3a, a shift towards lower temperatures is detected as the barium content is lowered, so the samples become more easily reducible. When comparing the reduction profiles for BxM samples with the corresponding Mn_2_O_3_ and MnO_2_ references, it is evident that the reduction processes involving Mn (IV)/Mn (III) are favored as the Ba content decreases. This trend should be related to the improved mobility of the ions located in the network (that facilitates the redox processes), which is caused not only by the existence of cation vacancies (due to the decrease in the barium content), but also by the formation of oxygen vacancies, which occurs to ensure the electroneutrality [64].

The experimental H_2_ consumption, estimated using the area under the H_2_ consumption profiles (and CuO as reference sample), is compared in Figure 3b with the nominal hydrogen consumption calculated considering either Mn (IV) (green line) or Mn (III) (red line) as the unique manganese oxidation states. As all experimental values are closer to the nominal value corresponding to Mn (III), it is suggested that Mn (III) is the main oxidation state of manganese in the bulk, contrary to what is observed on the surface according to the XPS results. Thus, these charge compensation mechanisms are suggested to take place only on the surface of the samples. In addition, some experimental points are below the theoretical line for Mn (III), which means that the total reduction in the bulk Mn is not being achieved. In fact, only the B0.7M sample achieves the 100% of Mn reduction.

Based on reactions (2) and (3), the amount of oxygen consumed during the H_2_-TPR experiments was calculated and is presented as the data collected in Table 3. Using these data and the total amount of oxygen available in the perovskites (as calculated from the molecular formula), the percentage of oxygen consumed during H_2_-TPR tests has been estimated. According to these data, the percentage of oxygen consumed increases with the decrease in the Ba content, caused by the improved ionic mobility and the increase in the amount of Mn (IV).

**Figure 4:**
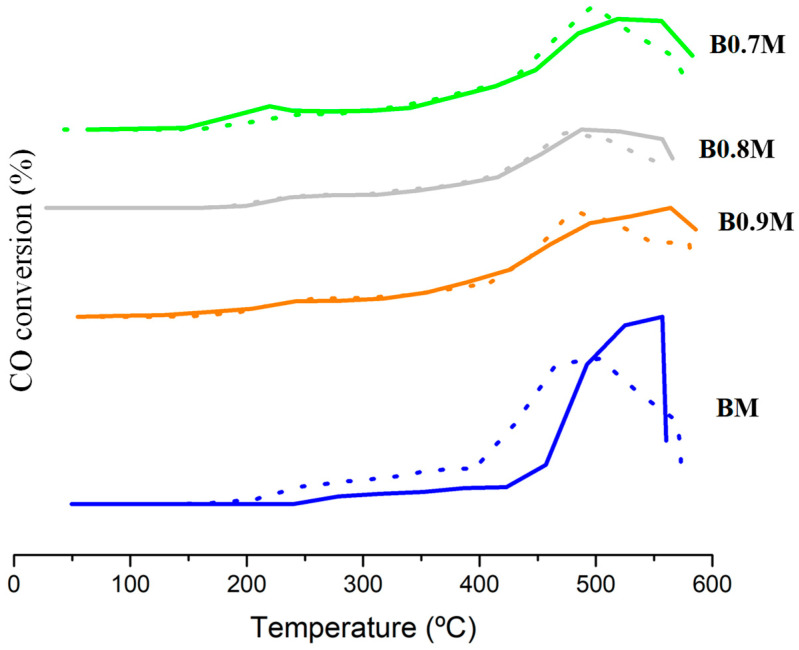
CO conversion profiles of the first (solid lines) and the second cycle (dotted lines) of CO-TPR tests for BxM samples.

CO-TPR experiments were also performed to obtain information about the interaction of CO with the samples. The profiles corresponding to the CO conversion percentage, in Temperature-Programmed conditions and inert atmosphere, are shown in Figure 4 as a function of temperature (solid line for first cycle and dotted line for second one) for all BxM perovskites. During these tests, as oxygen is not available in the reaction atmosphere, the CO will be oxidized using exclusively the oxygen evolved from perovskites. All BxM samples are able to oxidize CO even though the percentages of CO conversion are low (below 10%). Additionally, in the second CO-TPR tests, the temperature for the maximum CO conversion is shifted toward lower values with respect to the first cycle for all samples; so, the oxygen employed for the CO oxidation appears to be available at lower temperatures during the second cycle. The amount of oxygen involved in the CO-TPR test, calculated from CO conversion data using the CO oxidation reaction (CO + ½ O_2_ → CO_2_), and the corresponding percentage in regard to the total amount of oxygen in the perovskite (calculated based on the perovskite formula Ba_x_MnO_3_) have been included in Table 4. Note that, as expected due to the low CO conversion values, the percentages of O_2_ involved during the CO-TPR tests are low (around 25%, except for B0.8M), suggesting that the catalytic performance of the BxM samples for CO oxidation is highly dependent on the presence of oxygen in the reactant mixture. During the CO-TPR test, that is in the absence of oxygen, CO molecules should be adsorbed on the surface metal active sites [65,66], as the CO adsorption on oxygen vacancies implies a CO activation that is less effective than the activation on the metal active sites because the backdonation from oxygen vacancies to antibonding CO molecular orbitals does not take place [67,68]. However, despite the availability of metal active sites for CO adsorption and activation, the low CO conversion values obtained appear to be a consequence of a low supply of oxygen from the samples. Among samples, B0.8M presents the lowest CO conversions, as this perovskite involves the lowest amount of oxygen. Note that this sample shows the highest percentage of Mn (III) on the surface (XPS) and the lowest reducibility (H_2_-TPR) of the BxM series.

**Figure 5:**
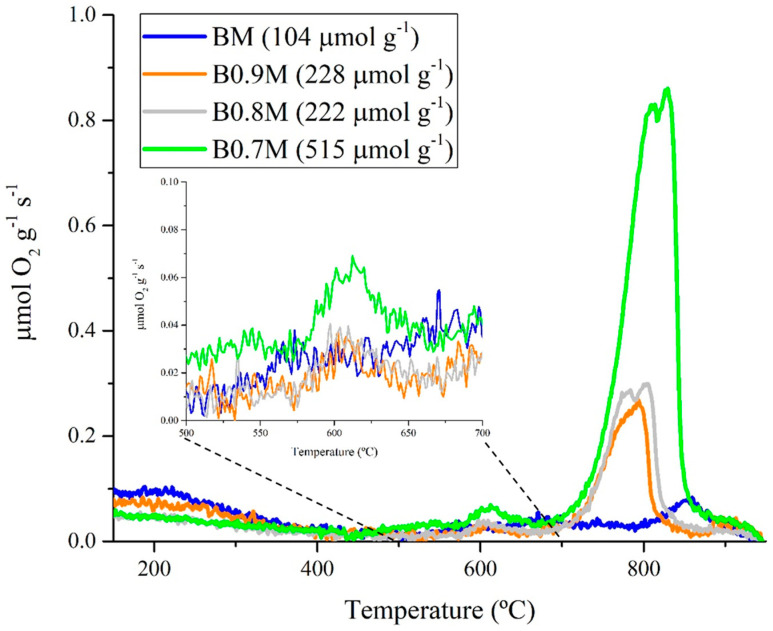
O_2_-TPD profiles for BxM samples.

To complete the perovskite characterization, the release of O_2_ from samples has been estimated by developing O_2_-TPD tests, and the corresponding O_2_ evolution profiles are shown in Figure 5. For perovskites, the following three peaks are usually found [69,70,71]: (i) a low temperature peak, between 150 °C and 350 °C, due to the desorption of oxygen adsorbed on surface vacancies (α-O_2_); (ii) a medium temperature peak from 350 °C to 700 °C, corresponding to the desorption of oxygen that comes from the defects that are adsorbed on the lattice (α’-O_2_); and (iii) a high temperature peak, over 700 °C, assigned to the release of lattice oxygen (β-O_2_), which depends on the reduction of Mn (IV) to Mn (III) and the presence of bulk oxygen vacancies, according to the Kröeger–Vink reaction (5) [72]. Thus, this high temperature peak informs us about lattice oxygen mobility through the perovskite network, which is related to the oxidation ability [69].

2Mn^x^_Mn_ + O^x^_O_ ⇆ 2Mn′_Mn_ + V^••^_O_ + 1/2O_2_ (5)

In reaction (5), Mn^x^_Mn_ and Mn′_Mn_ correspond to lattice Mn (IV) and Mn (III), respectively; additionally, O^x^_O_ is an oxygen atom placed in an O site and V^••^_O_ is an oxygen vacancy with a double deficiency of electrons. Note that, for all BxM perovskites, the O_2_ desorbed mainly corresponds to lattice oxygen (β-O_2_) even though a very small emission of both α-O_2_ (mainly for BM) and of α′-O_2_ (mainly for B0.7M) is also detected. The total amount of the oxygen evolved (data included in Figure 5) increases with the decrease in the barium percentage, suggesting that more oxygen vacancies are generated inside the structure (to neutralize the excess of negative charge due to the lack of Ba (II) cations) and that the amount of bulk Mn (IV) (whose reduction to Mn (III) promotes the oxygen emission) is higher. Thus, the mobility of oxygen through the structure is promoted for Ba-deficient perovskites, assuring a higher reducibility, as already deduced from H_2_-TPR results (Figure 3a). Furthermore, if the temperatures of oxygen emission are compared with those corresponding to the CO conversion during CO-TPR tests (Figure 4), it is suggested that α′-O_2_ species promote the oxidation of CO. So, the very low amount of α′-O_2_ evolved by perovskites appears to explain the low CO conversions during CO-TPR tests. Finally, it should be noted also that lower amounts of oxygen are released during O_2_-TPD experiments than during CO-TPR and H_2_-TPR tests; this is as expected, since CO and H_2_ are reducing agents that promote the oxygen emission [73]. Thus, the highest oxygen mobility of B0.7M would be linked to the highest proportion of Mn (IV) and to its high reducibility (see H_2_-TPR results). 

### 3.2. Catalytic Activity

**Figure 6:**
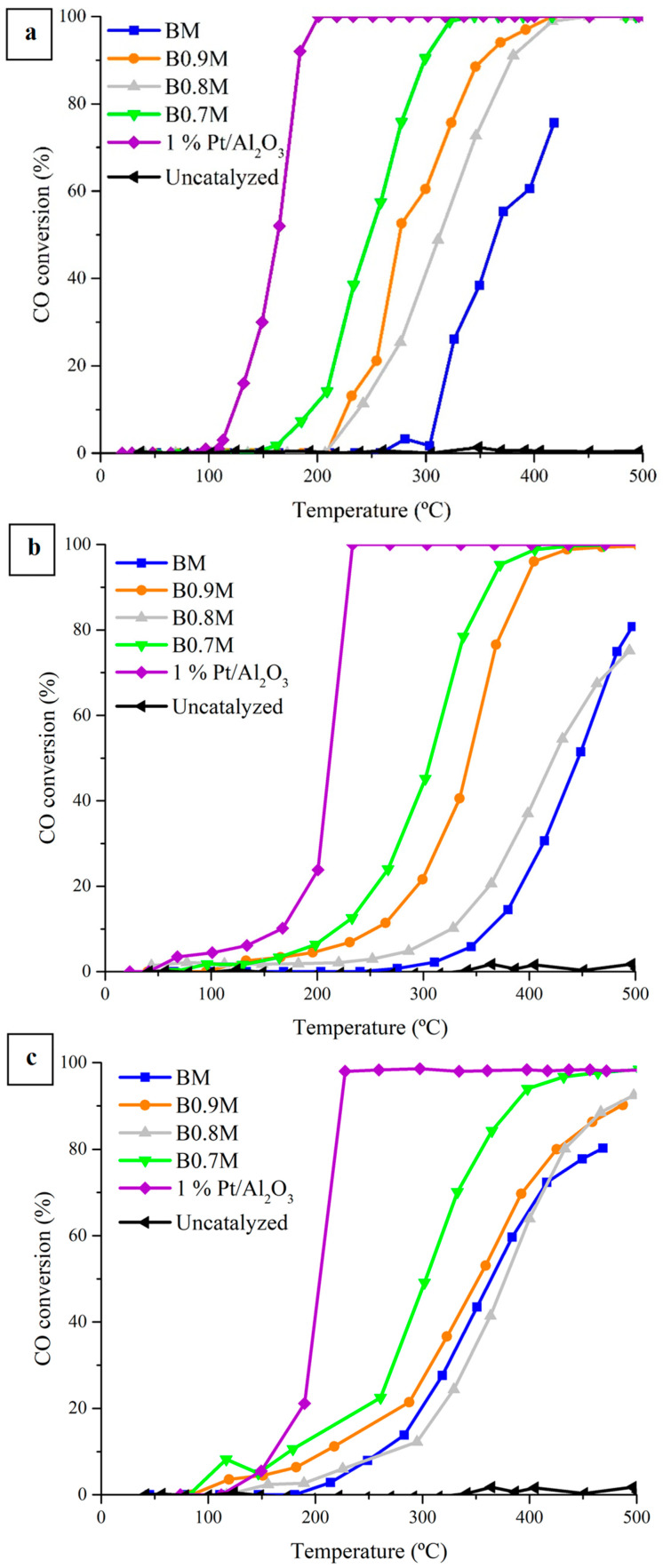
CO conversion profiles for BxM and 1% Pt/Al_2_O_3_ samples in the 0.1% CO/1% O_2_/He (**a**); 1% CO/1% O_2_/He (**b**); and 1% CO/10% O_2_/He (**c**) reactant mixtures.

Figure 6 shows the CO conversion profiles obtained under Temperature-Programmed Reaction conditions (CO-TPRe) for BxM samples in the three reaction gas mixtures tested, along with the corresponding to the 1% Pt/Al_2_O_3_ sample used as reference. The corresponding T_50%_ data (which is the temperature used to achieve a 50% CO conversion) are featured in Table 5.

These results reveal that all perovskites are active in regard to catalyzing the CO oxidation to CO_2_, as the reaction does not take place in the absence of a catalyst (see “Uncatalyzed” profiles). Moreover, most of the Ba-deficient perovskites overcome the catalytic performance of the BM sample, mainly due to the presence of a higher amount of oxygen vacancies on the surface (see XPS results), that act as active sites for CO oxidation, and an improved reducibility and lattice oxygen mobility (see H_2_-TPR and O_2_-TPD results). 

The comparison of T_50%_ values for the reactant atmospheres tested (Table 5) reveals that the catalytic performance of the B0.9M and B0.7M samples (especially the latter one) is not significantly affected by the increase in the O_2_ partial pressure; however, for the BM and B0.8M perovskites, a decrease in the T_50%_ is observed, and so the performance of these two samples is improved by an increase in the partial pressure of oxygen. However, the increase in the CO partial pressure originates a worse catalytic performance for all samples, as all the T_50%,CO_ values increase, with B0.7M being the least affected. 

Based on the literature [38,74,75,76,77], three mechanisms can drive the CO oxidation reaction in the presence of oxygen vacancies: the Langmuir–Hinshelwood (L-H), Eley–Rideal (E-R) and Mars–van Krevelen (MvK). Briefly, in the L-H mechanism, oxygen is firstly adsorbed on the oxygen vacancies; meanwhile, CO is adsorbed on the Mn sites, and both adsorbed molecules react for CO_2_ production. In the E-R pathway, O_2_ is adsorbed on the corresponding active sites and CO reacts directly from the gas phase. Finally, in the MvK mechanism, CO is adsorbed on the Mn sites, and these adsorbed species are subsequently oxidized using the lattice oxygen, being the generated oxygen vacancies refilled by taking oxygen from the reactant atmosphere. On the other hand, as commented above, the metal species are the main adsorption sites for CO [67,68], while oxygen vacancies play an essential role in O_2_ adsorption. However, computational studies [78] have demonstrated that O_2_ adsorption and activation can also occur on the metal active sites; therefore, a competition between CO and O_2_ by active sites could be taking place. Thus, for BM and B0.8M, a strong competition of CO and O_2_ by the active sites could be suggested since these samples present the highest values of ΔT_50%,CO_, as the active sites for CO should be more limited than oxygen active sites. Thus, BM and B0.8M show the worst catalytic performance as they present a lower amount of oxygen vacancies and Mn (IV) on the surface and a lower reducibility and oxygen mobility. In fact, B0.8M also features the worst performance during CO-TPR tests, which appears to be due to its low oxygen mobility and reducibility. Regarding B0.9M, it overcomes B0.8M, as it presents a higher reducibility and a slightly increased oxygen mobility. Finally, B0.7M is the most active perovskite from the BxM series, as it features the highest amount of oxygen vacancies on the surface and the highest reducibility and lattice oxygen mobility. Additionally, considering that C. Drosou et al. proposed that the improved lattice oxygen mobility in La_1−x_Sr_x_MnO_3_ perovskites (due to the lower positive charge in the A-site) favored the contribution of the MvK mechanism for CO oxidation reaction [76], it is suggested that the MvK mechanism could have a higher contribution in the global reaction pathway for the B0.7M perovskite. 

**Figure 7:**
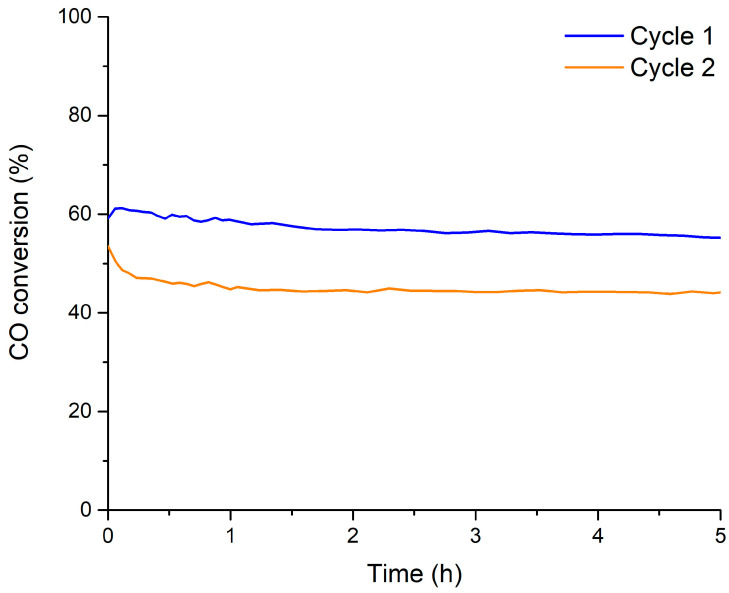
CO conversion profiles of B0.7M at 300 °C in the 1% CO/He reactant mixture.

Finally, a deeper study of the catalytic performance of the B0.7M sample has been carried out by developing two consecutive reactions at 300 °C (5 h) in 1% CO/1% O_2_/He reactant mixture (see Section 2 for more details), and the corresponding CO conversion profiles are featured in Figure 7. The CO conversion remains almost constant during the 5 h of reaction time of the two cycles, with the percentage for the first cycle being even higher than those previously measured in CO-TPRe conditions at 300 °C. However, for the second cycle, the CO conversion percentage is lower and more similar to the percentage observed in CO-TPRe conditions (Figure 6b). 

**Figure 8:**
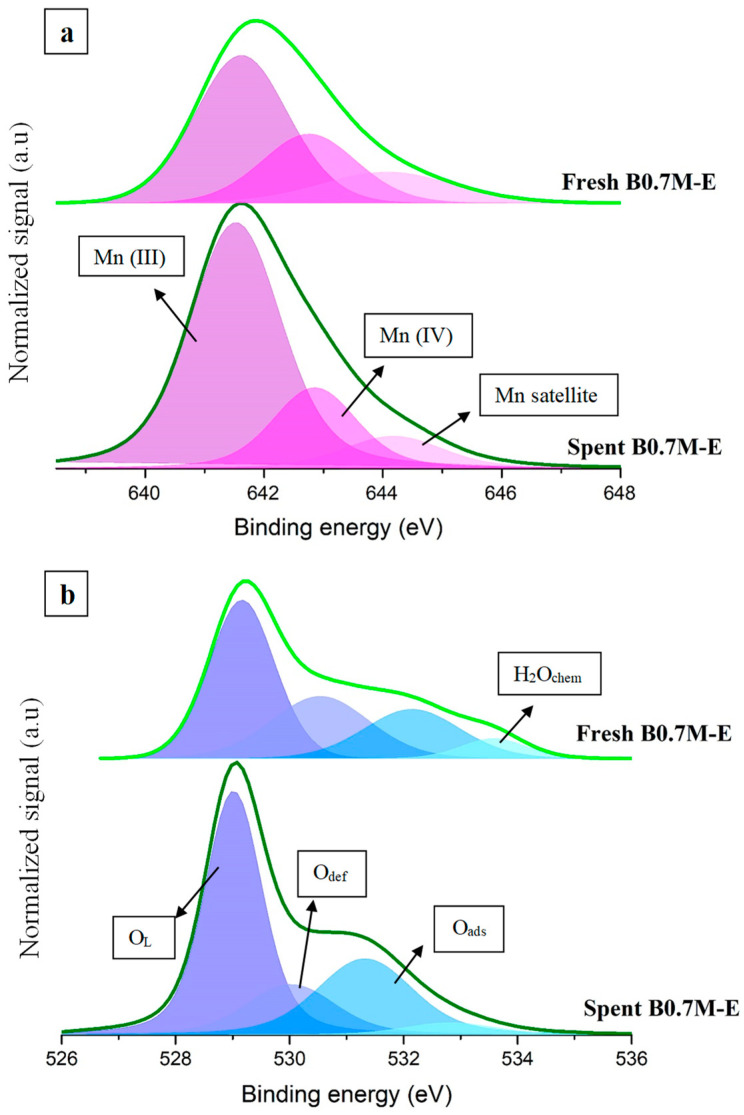
XPS spectra of the Mn 2p_3/2_ (**a**) and O 1s (**b**) transitions for the fresh and the spent B0.7M samples.

In order to explain the performance of the B0.7M sample during isothermal reaction cycles at 300 °C, the spent catalyst was characterized by XPS. Figure 8 shows the XPS spectra (Mn 2p_3/2_, Mn 3p and O 1s transitions) of the spent and the fresh samples, being the data of Mn (IV)/Mn (III) and O_L_/(Ba + Mn) ratios, and of the BE for the Mn 3p transition, collected in Table 6. The analysis of these data reveals that the decrease in the CO conversion in the second cycle appears to be related to the decrease in the Mn (IV)/Mn (III) ratio; so, a lower amount of Mn (IV) would be present on the surface, which implies a lower reducibility and oxygen mobility. The shift of the Mn 3p signal towards lower binding energies also confirms that the amount of Mn (III) is higher for the spent sample than for the fresh one. 

## 4. Conclusions

In this work, a series of Ba_x_MnO_3_ (x = 1, 0.9, 0.8 and 0.7) samples were synthesized, characterized and tested for CO oxidation reaction under simulated gasoline and diesel exhaust conditions. The key conclusions drawn are the following:

- The synthesis procedure allowed for achieving a perovskite-type structure, as confirmed by XRD data.
- XPS data substantiated the coexistence of a Mn(IV)/Mn(III) pair on the surface of the samples and, also, the presence of surface oxygen vacancies, being particularly prominent for B0.7M.
- The presence of oxygen vacancies in the Ba-deficient perovskites lattice allows us to increase the reducibility of samples and the oxygen mobility.
- The higher amount of oxygen vacancies and the improved reducibility of the Ba-deficient samples boosted the catalytic activity for the CO oxidation reaction of BM, even though the 1% Pt/Al_2_O_3_ reference sample still presents the best performance.
- B0.7M is the most active catalyst from the BxM series, as it presents the highest amount of oxygen vacancies on the surface (XPS), the highest reducibility (H_2_-TPR) and lattice oxygen mobility (O_2_-TPD and CO-TPR).

## Figures and Tables

**Table 1:** Chemical composition, specific surface area and XRD data of BxM catalysts.

Molecular Formula	Nomenclature	BET Surface Area (m^2^ g^−1^)	Chemical Composition (%)			Cell Parameters (Å) ^1^		Average Crystallite Size (nm)	Lattice Strain
			Ba	Mn	O	a	c		
BaMnO_3_	BM	9	62	24	14	5.7	4.8	24.8	3 × 10^−4^
Ba_0.9_MnO_3_	B0.9M	8	58	27	15	5.7	4.8	27.2	3 × 10^−5^
Ba_0.8_MnO_3_	B0.8M	10	56	29	15	5.7	4.8	26.7	4 × 10^−5^
Ba_0.7_MnO_3_	B0.7M	11	55	30	15	5.7	4.8	26.2	2 × 10^−4^

^1^ Considering that the relationship between the cell parameters in the hexagonal crystal system is *a* = *b* ≠ *c*, only *a* and *c* parameters are included in this table.

**Table 2:** Mn and O XPS characterization data of BxM samples.

Catalyst	Mn (IV)/Mn (III)	Mn (III)_s_ (%)	Mn (IV)_s_ (%)	XPS O_L_/(Ba + Mn) (Nominal)
BM	0.2	20	4	1.2 (1.5)
B0.9M	0.4	19	8	1.0 (1.6)
B0.8M	0.4	21	8	1.0 (1.7)
B0.7M	0.5	20	10	1.1 (1.8)

**Table 3:** O_2_ consumption during H_2_-TPR experiments.

Catalyst	Amount of O_2_ Consumed (μmol O_2_ (g cat)^−1^)	Percentage of O_2_ Consumed (%)
BM	845	14
B0.9M	1040	16
B0.8M	1030	15
B0.7M	1330	18

**Table 4:** Data for O_2_ involved in CO-TPR tests for BM and BxM perovskites (calculated employing the CO conversions and assuming the absence of parallel reactions).

Catalyst	O_2_ Involved (μmol O_2_ (g cat)^−1^) ^1^		Percentage of O_2_ Involved (%) ^2^		CO Converted (%) ^3^	
	Cycle 1	Cycle 2	Cycle 1	Cycle 2	Cycle 1	Cycle 2
BM	1678	1855	27	30	8	9
B0.9M	1601	1501	24	23	8	7
B0.8M	1164	1029	17	15	5	5
B0.7M	1763	1652	23	22	8	7

^1^ Calculated based on the percentages of CO conversion. ^2^ Calculated as: (μmol of O_2_ consumed/total amount of O_2_ in the perovskite) × 100. ^3^ Calculated as: (μmol of CO consumed during the experiment/total inlet amount of CO) × 100.

**Table 5:** T_50%_ data for BxM and 1% Pt/Al_2_O_3_ samples.

Catalyst	T_50%_ (°C)			ΔT_50%,CO_ (°C) ^1^	ΔT_50%,O2_ (°C) ^2^
	0.1%CO/1%O_2_/He	1%CO/1%O_2_/He	1%CO/10%O_2_/He		
BM	364	446	364	82	−82
B0.9M	276	343	352	67	9
B0.8M	313	423	377	110	−46
B0.7M	249	307	303	58	−4
1% Pt/Al_2_O_3_	164	212	204	48	−8

^1^ T_50%_ change after increasing the CO concentration from 0.1% to 1% (P_O2_ constant). ^2^ T_50%_ change after increasing the O_2_ concentration from 1% to 10% (P_CO_ constant).

**Table 6:** Mn and O XPS characterization data of the fresh and spent B0.7M samples.

Sample	Mn (IV)/Mn (III)	XPS O_L_/(Ba + Mn)	Mn 3p Position (eV)
Fresh B0.7M	0.5	1.1	50
Spent B0.7M	0.3	0.2	49

## Data Availability

Data are contained within the article.
